# Toward the Optimal Way to Assess Symptomatic Fatigue in Degenerative Cervical Myelopathy: Mixed Methods Survey Study

**DOI:** 10.2196/88794

**Published:** 2026-09-10

**Authors:** Timothy F Boerger, Alvaro Yanez Touzet, Julio C Furlan, Aria Nouri, Carl M Zipser, Brian K Kwon, Michael G Fehlings, James D Guest, Allan R Martin, Caroline Treanor, David B Anderson, Ellen Sarewitz, Lindsay Tetreault, James Harrop, Jefferson Wilson, Robert Chen, Vafa Rahimi-Movaghar, Nader Fallah, Julia Carter, Shirley Widdop, Shekar Kurpad, Bizhan Aarabi, Oliver Mowforth, Mark Kotter, Benjamin M Davies

**Affiliations:** 1 Joint Department of Biomedical Engineering Marquette University and the Medical College of Wisconsin Milwaukee, WI United States; 2 Scarborough General Hospital, York and Scarborough Teaching Hospitals NHS Foundation Trust York United Kingdom; 3 KITE Research Institute and Toronto Rehabilitation Institute University Health Network Toronto, ON Canada; 4 Department of Medicine Division of Physical Medicine and Rehabilitation University of Toronto Toronto, ON Canada; 5 Division of Neurosurgery Geneva University Hospitals Geneva Switzerland; 6 Spinal Cord Injury Centre Universitätsklinik Balgrist Balgrist, Zurich Switzerland; 7 Department of Orthopaedics International Collaboration on Repair Discoveries University of British Columbia Vancouver, BC Canada; 8 Division of Neurosurgery and Spine Program University of Toronto Toronto, ON Canada; 9 Neurological Surgery and the Miami Project to Cure Paralysis University of Miami Miami, FL United States; 10 Department of Neurological Surgery University of California, Davis Davis, CA United States; 11 School of Physiotherapy Royal College of Surgeons in Ireland Dublin Ireland; 12 Departments of Physiotherapy and Neurosurgery Beaumont Hospital Dublin Ireland; 13 School of Health Sciences Faculty of Medicine and Health The University of Sydney Sydney, New South Wales Australia; 14 Sydney Musculoskeletal Health Faculty of Medicine and Health The University of Sydney Sydney, New South Wales Australia; 15 Myelopathy.org Cambridge United Kingdom; 16 Department of Neurology Massachusetts General Hospital Boston, MA United States; 17 Department of Neurology Brigham and Women's Hospital Boston, MA United States; 18 Department of Neurosurgery Thomas Jefferson University Hospital Philadelphia, PA United States; 19 Division of Neurosurgery Department of Surgery St. Michael's Hospital Toronto, ON Canada; 20 Li Ka Shring Knowledge Institute St. Michael's Hospital Toronto, ON Canada; 21 Institute of Health Policy, Management and Evaluation Dalla Lana School of Public Health University of Toronto Toronto, ON Canada; 22 Division of Neurology University of Toronto Toronto, ON Canada; 23 Department of Neurosurgery Sina Trauma and Surgery Research Centre Tehran University of Medical Sciences Tehran Iran; 24 Praxis Spinal Cord Institute Vancouver, BC Canada; 25 Department of Neurosurgery Medical College of Wisconsin Milwaukee, WI United States; 26 Department of Neurosurgery University of Maryland School of Medicine Baltimore, MD United States; 27 Division of Neurosurgery, Department of Clinical Neurosciences University of Cambridge Cambridge, England United Kingdom

**Keywords:** degenerative cervical myelopathy, cervical spondylotic myelopathy, fatigue, patient-reported outcomes, validity, content validity, outcome assessment, psychometrics, mixed methods

## Abstract

**Background:**

Recent work has demonstrated that individuals with degenerative cervical myelopathy (DCM) often experience symptoms of fatigue that are underrepresented in the literature. Conversely, understanding fatigue is highly important to people living with DCM. The AO Spine RECODE-DCM (Research Objectives and Common Data Elements for Degenerative Cervical Myelopathy) study group recently determined that no suitable fatigue scales exist for DCM.

**Objective:**

An assessment of content validity of existing fatigue scales used in other populations was undertaken to determine whether any such scales are suitable for DCM.

**Methods:**

An expert panel of people with DCM was convened. The panel comprised members of the RECODE-DCM steering committee, and their DCM ranged in severity from mild to severe; all resided in the United Kingdom or the United States. They were presented with 19 instruments in use for other pathologies and developed a quantitative description of relevance to DCM. The committee scored each scale on clarity and comprehensiveness. We conducted a preliminary qualitative analysis of comments on the various attributes of each instrument to establish themes distinguishing high-scoring from lower-scoring instruments.

**Results:**

The highest-scoring scales were the (1) Modified Fatigue Impact Scale for Spinal Cord Injury (relevance=3.78; comprehensiveness=3.56; clarity=4), (2) Multidimensional Assessment of Fatigue (3.67, 3.56, and 3.89, respectively), and (3) Functional Assessment of Chronic Illness Therapy–Fatigue Scale (3.56, 3.56, and 3.89, respectively). Common themes used in discussing various instruments were sleep, pain, psychosocial factors, and employment.

**Conclusions:**

The Modified Fatigue Impact Scale for Spinal Cord Injury, Multidimensional Assessment of Fatigue, and Functional Assessment of Chronic Illness Therapy–Fatigue Scale appear to be the most appropriate and validated instruments for assessing fatigue in patients with DCM. These scales demonstrate strong psychometric properties while addressing the unique symptomatic profile and functional limitations characteristic of DCM, enabling more accurate measurement and clinical monitoring of fatigue in this patient population.

## Introduction

Degenerative cervical myelopathy (DCM) [1-4] is a common spinal cord condition whereby spinal intervertebral disc degeneration or hypertrophy and calcification of ligaments result in chronic, progressive compression of the spinal cord [5].

After championing by people with DCM, fatigue was identified as a core outcome for clinical trials on treatments for DCM by RECODE-DCM (Research Objectives and Common Data Elements for Degenerative Cervical Myelopathy), a multistakeholder global consensus process, to accelerate knowledge discovery in DCM [6,7].

Although fatigue has not been previously explored in DCM, there are several physiological underpinnings for elevated fatigue in people with DCM, including weakness, reduction of corticospinal drive to lower motor neurons, pain, medication-related somnolence, inflammation, spasticity, sarcopenia, and reduction in physical activity [8-12]. In addition, reduced functional capabilities, potential risk of injury with activities, and pain influencing sleep can reduce physical reserve and worsen fatigue.

Having identified a list of core outcomes (“what to measure”), RECODE-DCM went on to develop a minimum dataset that included a list of core measures for all clinical trials (“how to measure”) [13,14]. For outcomes not previously measured in DCM, potential tools in use for other clinical conditions were considered. However, by definition, this meant that their psychometric properties with respect to DCM were unknown. To inform their suitability, the face validity of such tools, including fatigue tools, was appraised by a panel of people with DCM [13-15].

Face validity is a simple method of appraising content validity as defined by the Consensus-Based Standards for the Selection of Health Measurement Instruments (COSMIN) framework [16-18]. Specifically, if items are not relevant, comprehensive, and comprehensible to the target population and the characteristic being studied, all other measurement properties (structural validity, internal consistency, and repeatability) will be affected [17]. Content validity is foremost in determining a tool’s appropriateness for use. COSMIN recommends that evaluation include persons with lived experience of the disease.

In this paper, we present the provisional assessment of content validity for measures of fatigue in DCM by a panel of people with DCM. Tools were short-listed from a systematic review [15]. This substudy was used to inform the development of the DCM minimum dataset. The results are described in detail, given that the management of fatigue is now a recognized and critical knowledge gap for people with DCM, to help inform future investigations. This aligns with the overall objectives of RECODE-DCM to help accelerate the efficient creation and translation of knowledge that can change outcomes [19].

## Methods

### Overview

Nine people with DCM were asked to rate the survey scales; all were members of the RECODE-DCM steering committee. RECODE-DCM is an international community working to accelerate knowledge discovery and translation that can improve outcomes in DCM. This original project was delivered in partnership with AO Spine, a global nonprofit organization for spine surgeons. RECODE-DCM now sits within Myelopathy.org, a global DCM charity. People with DCM were purposively recruited to participate in the RECODE-DCM steering committee from within the professional network of the RECODE-DCM principal investigators. As such, participants and researchers knew each other for at least 1 year prior to completing the survey. Expertise in people with DCM was defined as having a diagnosis of DCM while also serving on DCM consensus workgroups, managing support groups, engaging in research, being involved in the Myelopathy.org charity, and/or other patient advocacy efforts for DCM. Notably, the first author of this paper was a survey rater. His summary scores largely agreed with the prevailing views of the other raters. Scores including his data were highly correlated with scores excluding his data (*r*=0.98). Of the 9 people with DCM who rated the scales, 6 provided demographic information. Of these 6 individuals, 4 were female, 3 resided in the United Kingdom, and 3 resided in the United States. Their mean Modified Japanese Orthopaedic Association score was 11.00 (SD 4.05; range 7-17 out of 18 points), and the mean age was 53.38 (SD 12.14; range 33.17-69.98) years. The survey was administered between March 1 and 14, 2022.

The methods specific to this study have been previously reported elsewhere [14]. In brief, we previously established the core outcome set (COS) and core measurement set (CMS) for DCM. Between establishing the COS, which determined that fatigue, mental health, pain, and economic impact were “core” outcomes, and subsequently establishing the CMS, a systematic review was undertaken to identify questionnaire instruments related to fatigue used in research related to DCM [15]. As no suitable scale was identified, the systematic review was expanded to conceptually similar conditions to DCM that affect the nervous system (ie, traumatic spinal cord injury [SCI], fibromyalgia, and stroke) [15]. This was performed primarily to determine whether any scale was suitable for inclusion in the CMS. This resulted in a list of 19 fatigue scales potentially suitable for consideration. These scales were presented to people with DCM involved in the RECODE-DCM steering committee for their consideration of the suitability of each scale.

People with DCM were queried regarding their perceptions of the relevance, comprehensiveness, and clarity of the fatigue scales (Multimedia Appendix 1). Question items and responses were adapted from the COSMIN manual. Perceptions were scored on 4-point Likert scales ranging from 1 (low intensity of agreement) to 4 (high intensity of agreement). For the relevance item, 1 implied “no items are relevant,” and 4 indicated “all items are relevant.” Responses to the comprehensiveness question ranged from 1 (“key aspects are missing”) to 4 (“no key aspects are missing”). For clarity, 1 indicated “not clear,” and 4 indicated “very clear.” Operationally, we defined a mean score of 3.5 or more across raters as a “good” score for each domain. Additionally, people with DCM were asked to estimate how long (in minutes) each instrument would require for completion. This was considered of relevance to the project’s principal purpose of informing a minimum dataset for DCM research where, given a wide range of core outcomes, the potential burden on participants to complete all assessments would need to be balanced against its broader value for DCM research. Finally, a free-response question was posed to people with DCM to elicit comments on the perceived qualities of each instrument. This was intended to provide preliminary evidence on the perspectives of people with DCM with respect to the potential fatigue scales listed below. This evidence will be used to streamline future mixed methods studies. This cohort of individuals was derived largely from the United States and the United Kingdom. Following the scoring of each domain (eg, relevance, comprehensiveness, and clarity), scores across domains were averaged to obtain a summary score from each person for each instrument.

In total, people with DCM were asked to evaluate and provide scores for 19 fatigue-related tools. These tools were the visual analog scale for fatigue [20], Fatigue Assessment Instrument [21], Modified Fatigue Impact Scale for Spinal Cord Injury (MFIS-SCI) [22], 36-item Short Form Health Survey (SF-36) fatigue subscale [23], Functional Assessment of Chronic Illness Therapy–Fatigue Scale (FACIT-F) [24], Multidimensional Assessment of Fatigue (MAF) [25], Chronic Fatigue Syndrome Activities and Participation Questionnaire [26], Fatigue Symptom Inventory (FSI) [27], Checklist Individual Strength (CIS) [28], FibroFatigue Scale (FFS) [29], Bristol Rheumatoid Arthritis Fatigue Multidimensional Questionnaire [30], Multidimensional Daily Diary of Fatigue–Fibromyalgia–17 (MDF-Fibro-17) [31], Fatigue Assessment Scale [32], numeric rating scale for fatigue [33], Chalder fatigue scale [33], Brief Fatigue Inventory (BFI) [34], Fatigue Impact Scale (FIS) [35], Multidimensional Fatigue Inventory (MFI) [36], and Piper Fatigue Scale [37].

In addition to providing subjective scoring, respondents were asked to provide free-text responses via an online survey to discuss what they did or did not like about each tool. The survey was sent to participants via email by one investigator (BMD), a male trainee physician trained in qualitative methods. Data were collected virtually. Responses were collated in Microsoft Excel. Responses were read through and coded and recoded after a period of 1 month to arrive at an accurate and consistent representation of the data. Analyses were conducted by one investigator (TFB), a male PhD scientist with DSM trained in qualitative methods. Thematic analysis was used to inductively identify codes and themes in the written responses from participants. For example, comments about requiring naps during the day were coded as “nap,” and comments about disturbed sleep were coded as “sleep.” Subsequently, themes were identified from underlying codes and stored in Microsoft Excel. As such, codes broadly related to the length, layout, or clarity of the instrument were themed as “structure.” Codes including words such as “psychosocial” and “socioemotional” were assigned to the theme “psychosocial.” Codes related to the length of recall were themed “recall.” Filler words such as “liked,” “mentions,” and “perhaps” were discarded. All the free responses were then entered into word cloud–generating software that extracted the top 10 most frequent words, which is a validated method [38,39]. The first author analyzing the results continuously reflected on whether results were due to his own biases, including that he is a person with DCM, and he discussed underlying responses, codes, and thematic results with the second and last authors. The results were compared to the published COS for DCM. Participants were invited to comment on the results and were included as authors if they desired. This study was intended to inform future qualitative focus groups and larger-scale qualitative survey studies.

### Statistical Analysis

Given the qualitative-to-semiquantitative nature of this study, no formal hypothesis testing was undertaken. Nevertheless, descriptive and summary statistics were calculated in Microsoft Excel.

### Ethical Considerations

Ethics approval was obtained from the University of Cambridge (HBREC2019.14) in accordance with the Declaration of Helsinki.

## Results

### Overview

**Figure 1:**
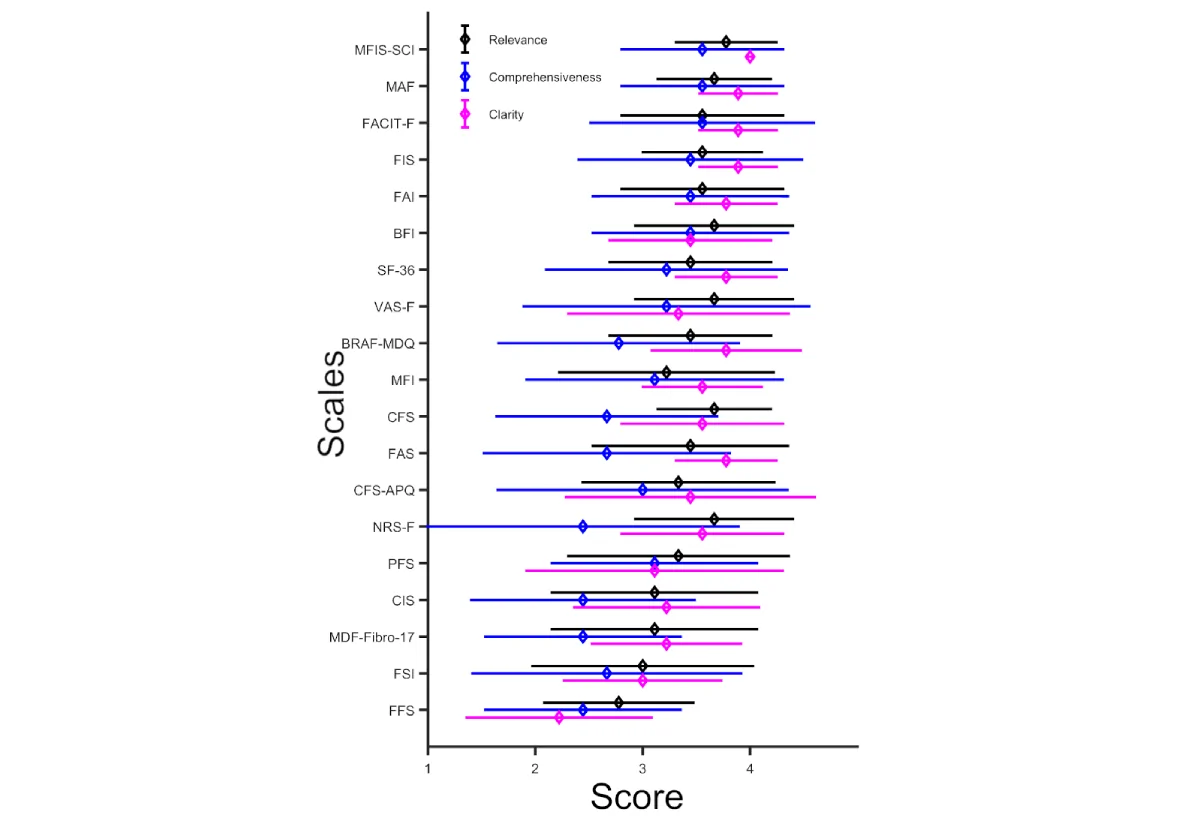
Content validity scores for each fatigue scale. Scores are provided for clarity (comprehensibility), comprehensiveness, and relevance to degenerative cervical myelopathy. Scales are ordered by the average sum of scores. BFI: Brief Fatigue Inventory; BRAF-MDQ: Bristol Rheumatoid Arthritis Fatigue Multidimensional Questionnaire; CFS-APQ: Chronic Fatigue Syndrome Activities and Participation Questionnaire; CFS: Chalder fatigue scale; CIS: Checklist Individual Strength; FACIT-F: Functional Assessment of Chronic Illness Therapy–Fatigue Scale; FAI: Fatigue Assessment Instrument; FAS: Fatigue Assessment Scale; FFS: FibroFatigue Scale; FIS: Fatigue Impact Scale; FSI: Fatigue Symptom Inventory; MAF: Multidimensional Assessment of Fatigue; MDF-Fibro-17: Multidimensional Daily Diary of Fatigue–Fibromyalgia–17; MFI: Multidimensional Fatigue Inventory; MFIS-SCI: Modified Fatigue Impact Scale for Spinal Cord Injury; NRS-F: numeric rating scale–fatigue; PFS: Piper Fatigue Scale; SF-36: 36-item Short Form Health Survey; VAS-F: visual analog scale for fatigue.

When pooled across all scores from all participants for all instruments, the median summary score was 3.67 (IQR 2.83-4), indicating that raters scored all instruments rather well. Four instruments had a between-participant average of more than 3.67 (MFIS-SCI, FACIT-F, MAF, and Fatigue Assessment Instrument). Four of the scales received a summary score below 3, specifically the FFS (summary score=2.48, SD 0.60), FSI (summary score=2.89, SD 0.91), MDF-Fibro-17 (summary score=2.93, SD 0.76), and CIS (summary score=2.93, SD 0.76). Generally, across scales, comprehensiveness was scored lower than relevance and clarity. Scores for the various scales are shown in Figure 1. The frequency of themes with respect to various scales is visually presented in Multimedia Appendix 2.

### Time to Complete

Of the top-scored scales, the mean times to complete were approximately 6 minutes (MFIS-SCI: mean 6.00, SD 4.03 minutes; FACIT-F: mean 7.56, SD 3.97 minutes; MAF: mean 6.67, SD 3.46 minutes). Thus, most scales could easily be administered without adding substantial testing burden in many clinical trials.

### Preliminary Qualitative Analysis of Content Validity

In total, 26 codes were generated from responses that were mapped to 7 themes, as can be observed in Table 1. One prevailing theme was an insufficient discussion of sleep. This was noted in regard to the FIS, MFIS-SCI, MAF, BFI, and MFI. Regarding the FIS some specific comments included the following:

...the problem with the fatigue I experience is that I have to sleep during the day 3 - 4 hours & the sleep I have at night is never refreshing & often disturbed.

Participant 7

**Table 1:** Primary codes and themes generated from the raw responses.

Theme	Codes
Structure	Clarity Relevance Understandable Thoroughness Ease Time requirements Duration of impact Reorganization Uninteresting
Physical function	Physical function Muscle weakness
Pain	Pain Discomfort Sleep Tiredness Nap
Psychosocial	Mood Distress Stigma Social effects Relationships Driving ability Sexual activity
Employment	Employment
Recall	Recall

Another respondent suggested that questions be added to the FIS and MFIS-SCI on requiring naps, such as “Do you require a nap during the day?” (participant 9). Regarding the MFIS-SCI, one responder said the following:

The impact of sleep or sleep disturbance is missing.

Participant 7

Another comment noted that the BFI “doesn’t include pain or sleep” (participant 7). On the other hand, the FACIT-F was commended for discussing sleep:

I like this one even better because it mentions the socioemotional impact of illness on yourself & the family & mentions sleep. It mentions pain too.

Participant 7

Other common themes were related to the effects of pain on fatigue and of fatigue on employment status. For example, the MAF, MDF-Fibro-17, MFI, CIS, and FFS were criticized for not accounting for the effects of pain on fatigue and/or of fatigue on employment status. The FACIT-F and MFIS-SCI, however, were commended for mentioning pain. Regarding the MFIS-SCI, it was said that “it discusses physical discomfort which is good” (participant 7).

Similarly, mood and psychological state was also a topic frequently commented on. The FACIT-F and SF-36 were commended for discussing “socioemotional impact.” Other scales, such as the Fatigue Assessment Scale and CIS, were criticized on this topic.

Other frequent comments related to the topic of aesthetics and setup or structure of the survey. This included comments such as “Far too detailed” (participant 3), “Feels like duplicate questions” (participant 5), and “It would take me [a long] time to recollect all the information it asks for” (participant 7) for the FSI and “A bit long winded for a person with DCM to fill out” for the BFI. This topic also included comments that the responder was “distracted/bothered by the layout or dark highlighted bars” (participant 4) for the BFI.

Another common topic of comment was on the duration of recall required from each scale. For example, scales that asked about symptoms within a 24-hour period or “right now” were viewed unfavorably compared to those that asked about symptoms within a 2- to 4-week period. For example, regarding the BFI, participant 8 commented that “24 hours seem like such a short time frame.” Another participant wrote the following regarding the BFI:

I am not sure 24-hour snapshot is sufficient. We have good and bad days.

Participant 9

This sentiment was related to views that DCM is variable and that too brief a recall period would fail to capture the worst days. For example, regarding the Chalder fatigue scale, one person wrote the following:

This is a months snapshot, this one works well.

Participant 9

### Common Criticisms of Poor- vs High-Performing Scales

In analyzing the criticisms of the bottom 3 scales in terms of performance, length and detail, format, and extraneous concepts were frequently noted for the FSI, CIS, and FFS. For example, criticisms of the FFS frequently included its long, poorly designed layout. Comments on the CIS included “Not as easy as the other formats” (participant 2) and “confusing—the scoring” (participant 4). Similarly, comments on the FSI included “Misses out on the physical fatiguability possible in DCM” (participant 1), “far too detailed” (participant 3), “would dwell on these” (participant 4), and “It would take me a long time to recollect all the information it asks for” (participant 7). Conversely, despite sometimes being criticized for being long, the top-scoring scales were described as more “clear” but also “relevant,” “comprehensive,” and “to the point.” Regarding the MFIS-SCI, comments included “Nicely broken down and helpful” (participant 4), “clearer questions and less bothersome” (participant 5), and “I like this scale—it seems comprehensive” (participant 7). Similarly, on the FACIT-F, one person responded that “this one seems more well-rounded” (participant 8). Regarding the MAF, one person responded that “I found this instrument a bit more to the point” (participant 5).

## Discussion

### Principal Results

The primary purpose of this study was to provide preliminary content validation of candidate fatigue scales existing in the current literature, and our primary finding was that the MFIS-SCI, MAF, and FACIT-F demonstrated good content validity. These scales could represent starting points to implement a fatigue scale into use in research in DCM. Our results further suggest that any future development of fatigue scales specific to DCM should incorporate assessments of sleep, effects of pain on fatigue, psychosocial factors such as stress due to having a chronic condition, and impact of fatigue on employment status. Alternatively, additional assessment scales for pain (eg, Neck Disability Index, Brief Pain Inventory, and numeric rating scale for pain), employment status, and mental health could supplement deficiencies in assessments of fatigue on these factors. These are in addition to assessments of lower-extremity fatigability (such as the 6-minute walk test) that have been shown to differ in healthy controls [40]. Furthermore, future studies examining the impact of symptomatic fatigue and neuromuscular fatigability on quality of life are needed.

### Comparisons to Prior Work

As noted above, fatigue was identified as a core outcome of DCM; however, we have little understanding of its biological basis in DCM. This presents several issues related to measurement validity, and we were previously unable to identify any scales with sufficient scientific evidence to be included in the CMS [13,15]. People with DCM have identified sleep, pain, employment status, and mental health as common critical components of fatigue in DCM. While the overall relationship between many of these and fatigue is poorly understood (and this includes the causal direction), we discuss them subsequently and present a hypothesized formative model of poor sleep, pain, and psychosocial factors driving fatigue, with employment status likely a consequence of fatigue.

The MFIS-SCI was the least surprising scale to be relevant to people with DCM as it is drawn from a highly similar clinical population (eg, SCI vs DCM) rather than chronic fatigue syndromes. These factors suggest that the MFIS-SCI is the most likely to effectively translate to a DCM population. The Modified FIS is also validated in multiple sclerosis and is designed to capture the “impact” of fatigue [41,42]. In comparison, the MAF was also designed to capture the “impact” of fatigue but structures questions as they relate to specific activities. The MAF does not cover cognitive aspects of fatigue (which the MFIS-SCI does); therefore, it is possible that people with DCM viewed the MAF as incomplete [25]. Additionally, the FACIT-F contains questions about feeling ill, feeling nauseous, side effects of treatment, and worries about death, which may have resulted in views that it is less relevant than the MAF or MFIS-SCI [24]. The FACIT-F, however, included more items directed at psychosocial factors. It is possible that the length of the FACIT-F (40 items in total) and the items that may be viewed as less relevant offset the effects of including more psychosocial items. This phenomenon may relate to the respective origins of the MAF (rheumatoid arthritis) and FACIT-F (patients with cancer).

### Critical Themes

#### Sleep

Sleep disturbances reportedly affect approximately 70% of people with DCM [43] and are also common in individuals who sustained a traumatic SCI. The consequences of disruption to the circadian system and sleep can be profound and include myriad metabolic ramifications with widespread health consequences [44]. Physiological changes associated with this theme have been demonstrated in individuals with spinal cord lesions [45,46] but not yet studied in DCM. Thus, a heightened interest in this by the respondents is unsurprising. Sleep disturbances can be profoundly disruptive to health-related quality of life and limit social participation and employability [47]. A recent investigation of sleep deprivation in DCM identified worse depression scale scores, a lower Modified Japanese Orthopaedic Association score, chronic shoulder joint pain, smaller spinal cord area, and decreased cervical range of motion as independent risk factors for sleep disturbance [43]. Critically, sleep disturbances are also intimately coupled with other themes discussed below, such as depression and pain.

To that end, the Neck Disability Index includes an item on the relationship between neck pathology and impaired sleep. Nevertheless, only a handful of studies have examined the relationship between DCM and poor sleep. As such, future research is needed to understand the potential impact of disordered sleeping on fatigue and functional impairments in people with DCM.

#### Pain

The importance of the role of pain on fatigue is unsurprising. Previous works have determined that pain is a key recovery priority of people with DCM. In people with DCM who have pain, pain can be highly disabling, widespread, and either musculoskeletal or neuropathic in origin [9]. This mirrors other conditions such as fibromyalgia, where pain and fatigue are coupled.

On the basis of prevalence and severity scores for pain obtained using the Neck Disability Index, pain may be present in approximately 80% of patients with DCM preoperatively. Moreover, of those who have pain, approximately 80% have pain of moderate severity or worse [9,48], and approximately 50% experience pain that substantially impacts major life events [49]. Importantly, pain is more frequent in female individuals and in patients aged 57 years or younger, with a BMI of 27 kg/m^2^ or more, and with gastrointestinal and rheumatological diseases [48]. Moreover, DCM, while known to cause frequent neck pain and occasional headaches, can also have a widespread impact on all 4 limbs, the back, and the abdomen [9]. Pain is commonly rated as interferential and weakly to strongly negatively associated with quality of life [9]. Beneficially, surgical interventions appear to reduce Neck Disability Index scores and transition patients away from being classified as having high-impact chronic pain [48,50].

#### Psychosocial

Mental health is known to be substantially compromised in people with DCM [51] and commonly associated with other factors such as pain and fatigue [9], as highlighted in a recent focus group for people with DCM [52]. Indeed, the prevalence of depression in people with DCM was estimated to be 37%, close to that among people with lumbar disc herniation [53]. Fortunately, mental health–related symptoms appear to improve following surgical decompression [54]. Having described the mental health experiences of people with DCM, the interrelationships among mental health, pain, and fatigue are discussed in the section below on the biopsychosocial model.

#### Employment

In other conditions, fatigue is frequently negatively associated with employment outcomes. For example, a recent systematic review on survivors of cancer identified 38 studies examining fatigue in the context of employment and productivity and concluded that employment status and productivity were both negatively affected by fatigue [55]. Similarly, negative associations between increased fatigue and employment outcomes have classically been documented in fibromyalgia; multiple sclerosis; and, more recently, COVID-19. Conversely, recently, fatigue has been reported as more of a barrier to employment for people with multiple sclerosis than those with SCI [56]. It is presently unclear how strongly fatigue may impact employment status and productivity in people with DCM. As other scales may overrepresent the importance of fatigue, this would further support the adoption of SCI-specific fatigue scales (eg, the MFIS-SCI), except that the MFIS-SCI does not explicitly mention employment [22]. The MFIS-SCI does discuss more broadly the impact of fatigue on tasks; physical activities; social activities; and lifestyles, in which respondents may include employment. This was not discussed by our panel of people with DCM with lived experience. Thus, broader consensus on the applicability of these questions to employment is unknown. Further research is therefore necessary to identify whether these items of the MFIS-SCI are associated with employment status specifically in people with DCM.

#### Structure

There were frequent criticisms of the length, layout, or clarity of the surveys that were evaluated; this speaks to the overall testing burden on the participants to complete the surveys. Surveys that are overly long or convoluted will be cognitively fatiguing. If future instruments are developed to assess fatigue tailored to DCM, more focused questionnaires are likely needed. Similarly, concise, easily understandable questions arranged in an aesthetically logical and appealing way will be critical. In that vein, “clarity” may have a protective effect on testing burden. That is, the top-scoring scales, even though they were sometimes lengthy, scored well in part because, as respondents described them, they were clear and “to the point.” This would suggest that participants may be willing to take part in longer scales (more questions) if those scales are well written such that there is a lower cognitive demand per question.

### Biopsychosocial Model

**Figure 2:**
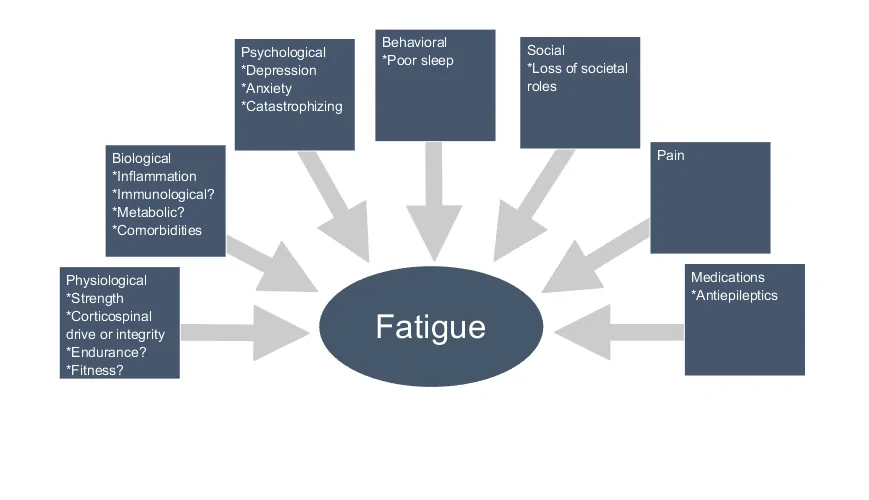
Biopsychosocial model applied to fatigue. This figure highlights the various biological, psychological, and social factors related to fatigue relevant to degenerative cervical myelopathy.

**Figure 3:**
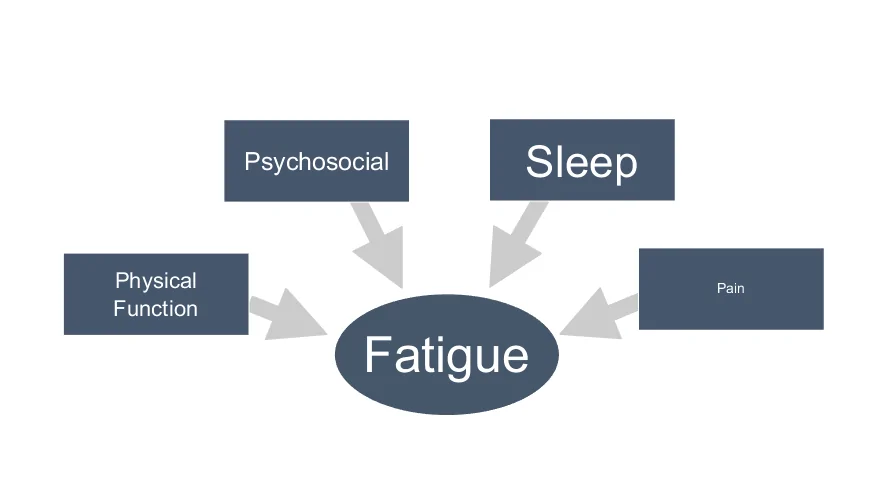
Biopsychosocial model weighted by participant preferences from the word cloud.

Improving our understanding of the pathophysiology of DCM is critical. Similarly, furthering our understanding of the pathophysiological mechanisms leading from spinal cord compression to symptoms such as fatigue will guide novel therapies to alleviate symptom burden in patients. As noted above, several of the themes identified by our expert panel of people with DCM are interrelated and consistent with other fatigue-related conditions. Themes such as sleepiness, pain, and mental health have been richly documented in fibromyalgia. It is not known, however, the extent to which the causal directionalities of these constructs are similar in DCM as they are in other conditions. Together, this cluster of symptoms is described using the biopsychosocial model (Figures 2 and 3), which has been applied to appreciate the interrelationship among pain, mental health, and fatigue in a variety of other conditions, including chronic fatigue syndrome, fibromyalgia, cancer, and traumatic brain injury. Importantly, there is preliminary evidence in people with DCM on a number of factors within the biopsychosocial model that contribute to fatigue (such as pain, inflammation, endurance, and mental health) [9,12,40,51,57].

In cancer, for example [58], fatigue was hypothesized to be caused by reductions in strength, endurance, cardiopulmonary fitness, and body composition as physiological factors. Similarly, fatigue was proposed to be directly caused by changes to immunological, metabolic, and endocrine functions and systemic inflammation. Fatigue was proposed to be caused via indirect pathways made up of psychological (eg, depression, anxiety, distress, and cognition), behavioral (eg, sleep quantity and quality and appetite), and social (eg, social interaction and positive reinforcement) factors. While this description from McNeely and Courneya [58] did not include pain, other relevant descriptions of biopsychosocial models of fatigue, such as for rheumatoid arthritis [59], have added pain to their model.

### Triangulation With Published Core Outcome Measures

Fatigue tools did not feature in the final DCM minimum dataset [13]. While the COS included fatigue, mental health, pain, and employment, none of these were explicitly included in the CMS. The measurement set pragmatically focused on one tool per domain (related group of core outcomes) and prioritized tools with a high-quality evidence base for use in DCM. This was intended to prevent measurement burden in trials. As such, the SF-36 version 2 was selected to address core outcomes such as mental health and employment [13,15]. The minimum dataset is designed to be used in all DCM trials. It is notable in our analysis, however, that the SF-36 was reported to be less clear, relevant, and comprehensive than the MFIS-SCI, MAF, and FACIT-F. As such, for dedicated investigations of fatigue in DCM, additional measurement tools will be required.

### Limitations

This study examined the perceptions of people living with DCM regarding the appropriateness of various existing self-reported outcome scales assessing fatigue. One limitation of this study was the small sample size, potentially resulting in a failure to reach saturation. However, our sample of people with DCM was recruited from multiple countries across Europe and North America and consisted of seasoned advocates who served on the RECODE-DCM steering committee and led peer support groups educating and advocating for people with DCM. Given these backgrounds, our panelists were well suited to gauge the included fatigue surveys based on their own experiences as well as common questions and symptom concerns raised by others. Additionally, we were unable to capture data on potential comorbidities from our panelists as this study was a retrospective secondary analysis. A follow-up, true qualitative study using focus groups capturing comprehensive data on comorbidities that could impact perspectives on fatigue should be conducted based on the short-listed scales to recruit additional opinions and control for confounding comorbidities. Nevertheless, our results largely agree with our previously published COS based on a modified Delphi process relying on the opinions of 113 people with DCM. This COS defined fatigue, mental health, pain, and economic impact as core outcomes. Another potential limitation is related to cross-cultural adaptability [60]. All scales were tested in English, and the participants surveyed in this study were all native English speakers. While English makes nuanced distinctions among words such as “sleepy,” “tired,” and “fatigued,” other languages make no distinction between some of these terms. For this reason, our recommendations should only be taken for English-based studies unless there is a validated version in a research study’s target language (eg, the Modified FIS). Finally, establishing the validity of an instrument encompasses numerous components. Other aspects of validity (eg, structural validity, convergent validity, internal consistency, and repeatability) remain to be established in DCM specifically for these scales.

### Conclusions

Fatigue is recognized as an important symptom of DCM, but it remains poorly understood. Existing measures are considered reasonable for use in DCM but need further field-testing. The MFIS-SCI, MAF, and FACIT-F were the preferred outcome measures according to the people with DCM who participated in this study. While these represent a starting point, panelists recognized limitations, and it is likely that dedicated measurement and/or additional measurement approaches are required to address fatigue in DCM.

## Data Availability

The datasets generated or analyzed during this study are available from the corresponding author on reasonable request.

## Appendix

Multimedia Appendix 1 Survey provided to raters.

Multimedia Appendix 2 Word cloud based on responses. Words were limited to the top 10 words with respect to frequency.

## Abbreviations

BFI: Brief Fatigue Inventory
CIS: Checklist Individual Strength
CMS: core measurement set
COS: core outcome set
COSMIN: Consensus-Based Standards for the Selection of Health Measurement Instruments
DCM: degenerative cervical myelopathy
FACIT-F: Functional Assessment of Chronic Illness Therapy–Fatigue Scale
FFS: FibroFatigue Scale
FIS: Fatigue Impact Scale
FSI: Fatigue Symptom Inventory
MAF: Multidimensional Assessment of Fatigue
MDF-Fibro-17: Multidimensional Daily Diary of Fatigue–Fibromyalgia–17
MFI: Multidimensional Fatigue Inventory
MFIS-SCI: Modified Fatigue Impact Scale for Spinal Cord Injury
RECODE-DCM: Research Objectives and Common Data Elements for Degenerative Cervical Myelopathy
SCI: spinal cord injury
SF-36: 36-item Short Form Health Survey
