# Supplementary material for: Toward the Optimal Way to Assess Symptomatic Fatigue in Degenerative Cervical Myelopathy: Mixed Methods Survey Study
Source: JMIR Form Res. 2026 Sep 10;10:e88794. doi: 10.2196/88794 (PMC13613046; doi:10.2196/88794)
Supplement: Supplementary file 1 [file formative_v10i1e88794_app1.pdf]

# Content validity of tools on 'fatigue'

* Indicates required question

## Background

Our core outcome set identified 'fatigue' as a core outcome.

We are now trying to decide which tools best evaluate fatigue for people living with DCM.

As this hasn't been measured before in DCM, we have looked at tools designed for other conditions.

## Purpose of this form

In this form, you will be asked about the relevance, clarity and comprehensiveness of 19 tools on fatigue.

We will also ask you how long you think each tool would take to fill in. Together, this will help us to understand how relevant and practical the tool would be for use in DCM

Each page presents one tool and four questions.

Please take a look at the tool and answer the four questions.

## Contact

Please email [redacted] with any questions.

To allow us to keep track of your answers, please enter your first name

1. First name *

## Tool #1

### Fatigue impact scale (FIS)

Please take a look at the tool below and answer the four questions:

**Because of my fatigue during the past 4 weeks**

| | | Never | Rarely | Sometimes | Often | Almost Always |
|---|---|---|---|---|---|---|
| 1. | I have been less alert. | 0 | 1 | 2 | 3 | 4 |
| 2. | I have had difficulty paying attention for long periods of time. | 0 | 1 | 2 | 3 | 4 |
| 3. | I have been unable to think clearly. | 0 | 1 | 2 | 3 | 4 |
| 4. | I have been clumsy and uncoordinated. | 0 | 1 | 2 | 3 | 4 |
| 5. | I have been forgetful. | 0 | 1 | 2 | 3 | 4 |
| 6. | I have had to pace myself in my physical activities. | 0 | 1 | 2 | 3 | 4 |
| 7. | I have been less motivated to do anything that requires physical effort. | 0 | 1 | 2 | 3 | 4 |
| 8. | I have been less motivated to participate in social activities. | 0 | 1 | 2 | 3 | 4 |
| 9. | I have been limited in my ability to do things away from home. | 0 | 1 | 2 | 3 | 4 |
| 10. | I have trouble maintaining physical effort for long periods. | 0 | 1 | 2 | 3 | 4 |
| 11. | I have had difficulty making decisions. | 0 | 1 | 2 | 3 | 4 |
| 12. | I have been less motivated to do anything that requires thinking | 0 | 1 | 2 | 3 | 4 |
| 13. | My muscles have felt weak | 0 | 1 | 2 | 3 | 4 |
| 14. | I have been physically uncomfortable. | 0 | 1 | 2 | 3 | 4 |
| 15. | I have had trouble finishing tasks that require thinking. | 0 | 1 | 2 | 3 | 4 |
| 16. | I have had difficulty organizing my thoughts when doing things at home or at work. | 0 | 1 | 2 | 3 | 4 |
| 17. | I have been less able to complete tasks that require physical effort. | 0 | 1 | 2 | 3 | 4 |
| 18. | My thinking has been slowed down. | 0 | 1 | 2 | 3 | 4 |
| 19. | I have had trouble concentrating. | 0 | 1 | 2 | 3 | 4 |
| 20. | I have limited my physical activities. | 0 | 1 | 2 | 3 | 4 |
| 21. | I have needed to rest more often or for longer periods. | 0 | 1 | 2 | 3 | 4 |

2. Relevance to DCM *

Are all items in this tool relevant to your experience of fatigue in DCM?

*Mark only one oval.*

| | 1 | 2 | 3 | 4 | |
|---|---|---|---|---|---|
| No i | ◯ | ◯ | ◯ | ◯ | All items are relevant |

3. Comprehensiveness *

   Does this tool miss any key aspects of your experience of fatigue in DCM?

   *Mark only one oval.*

| | 1 | 2 | 3 | 4 | |
|---|---|---|---|---|---|
| Key | ◯ | ◯ | ◯ | ◯ | No key aspects are missing |

4. Clarity *

   Was this tool clear to understand?

   *Mark only one oval.*

| | 1 | 2 | 3 | 4 | |
|---|---|---|---|---|---|
| Not | ◯ | ◯ | ◯ | ◯ | Very clear |

5. How long you do you think it would take to fill this tool? *

   Answer in minutes

   _______________________

6. Comments

   _______________________

## Tool #2

Modified fatigue impact scale for spinal cord injury (MFIS-SCI)
Please take a look at the tool below and answer the four questions:

Because of my fatigue during the past week…

| *Cognitive subscale* | *Never* | *Rarely* | *Sometimes* | *Often* | *Almost always* |
|---|---|---|---|---|---|
| 1. I have been less alert | 0 | 1 | 2 | 3 | 4 |
| 2. I have had difficulty paying attention for long periods of time | 0 | 1 | 2 | 3 | 4 |
| 3. I have been unable to think clearly | 0 | 1 | 2 | 3 | 4 |
| 4. I have been forgetful | 0 | 1 | 2 | 3 | 4 |
| 5. I have had difficulty paying attention for short periods of time | 0 | 1 | 2 | 3 | 4 |
| 6. I have had difficulty making decisions | 0 | 1 | 2 | 3 | 4 |
| 7. I have been less motivated to do anything that requires thinking | 0 | 1 | 2 | 3 | 4 |
| 8. I have had trouble finishing tasks that require thinking | 0 | 1 | 2 | 3 | 4 |
| 9. I have had difficulty organizing my thoughts when doing things | 0 | 1 | 2 | 3 | 4 |
| 10. My thinking has been slowed down | 0 | 1 | 2 | 3 | 4 |
| 11. I have had trouble concentrating | 0 | 1 | 2 | 3 | 4 |

Cognitive subscale score ________

| *Physical subscale* | *Never* | *Rarely* | *Sometimes* | *Often* | *Almost always* |
|---|---|---|---|---|---|
| 12. I have had to pace myself in my physical activities | 0 | 1 | 2 | 3 | 4 |
| 13. I have been less motivated to do anything that requires physical effort | 0 | 1 | 2 | 3 | 4 |
| 14. I have trouble maintaining physical effort for long periods | 0 | 1 | 2 | 3 | 4 |
| 15. I have trouble maintaining physical effort for short periods | 0 | 1 | 2 | 3 | 4 |
| 16. I have been physically uncomfortable | 0 | 1 | 2 | 3 | 4 |
| 17. I have been less able to complete tasks that require physical effort | 0 | 1 | 2 | 3 | 4 |
| 18. I have needed to rest more often or for longer periods | 0 | 1 | 2 | 3 | 4 |

Physical subscale score________

| *Psychosocial subscale* | *Never* | *Rarely* | *Sometimes* | *Often* | *Almost always* |
|---|---|---|---|---|---|
| 19. I have avoided/eliminated certain tasks, activities and lifestyles | 0 | 1 | 2 | 3 | 4 |
| 20. I have been less motivated to participate in social activities | 0 | 1 | 2 | 3 | 4 |
| 21. I have been limited in my ability to do things | 0 | 1 | 2 | 3 | 4 |

7. Relevance to DCM *

Are all items in this tool relevant to your experience of fatigue in DCM?

*Mark only one oval.*

| | 1 | 2 | 3 | 4 | |
|---|---|---|---|---|---|
| No i | ◯ | ◯ | ◯ | ◯ | All items are relevant |

8. Comprehensiveness *

Does this tool miss any key aspects of your experience of fatigue in DCM?

*Mark only one oval.*

| | 1 | 2 | 3 | 4 | |
|---|---|---|---|---|---|
| Key | ◯ | ◯ | ◯ | ◯ | No key aspects are missing |

9. Clarity *

Was this tool clear to understand?

*Mark only one oval.*

| | 1 | 2 | 3 | 4 | |
|---|---|---|---|---|---|
| Not | ◯ | ◯ | ◯ | ◯ | Very clear |

10. How long you do you think it would take to fill this tool? *

Answer in minutes

_______________________

11. Comments

_______________________
_______________________
_______________________
_______________________
_______________________

Tool #3

Functional assessment of chronic illness therapy: fatigue scale (FACIT-F)
Please take a look at the tool below and answer the four questions:

Click to view:
https://drive.google.com/file/d/1njeis9KJkJy_FcG9Jt2Wq_4TS63S2lid/view?usp=sharing

12. Relevance to DCM *

Are all items in this tool relevant to your experience of fatigue in DCM?

*Mark only one oval.*

| | 1 | 2 | 3 | 4 | |
|---|---|---|---|---|---|
| No i | ◯ | ◯ | ◯ | ◯ | All items are relevant |

13. Comprehensiveness *

Does this tool miss any key aspects of your experience of fatigue in DCM?

*Mark only one oval.*

| | 1 | 2 | 3 | 4 | |
|---|---|---|---|---|---|
| Key | ◯ | ◯ | ◯ | ◯ | No key aspects are missing |

14. Clarity *

Was this tool clear to understand?

*Mark only one oval.*

| | 1 | 2 | 3 | 4 | |
|---|---|---|---|---|---|
| Not | ◯ | ◯ | ◯ | ◯ | Very clear |

15. How long you do you think it would take to fill this tool? *

Answer in minutes

_______________________________

16. Comments

_______________________________
_______________________________
_______________________________
_______________________________
_______________________________

## Tool #4

Multidimensional assessment of fatigue (MAF)

Please take a look at the tool below and answer the four questions:

Click to view:
https://drive.google.com/file/d/1ICUI5SYVcsctw2f4VDaR_itmwAOQx2p1/view?usp=sharing

17. Relevance to DCM *

Are all items in this tool relevant to your experience of fatigue in DCM?

*Mark only one oval.*

| | 1 | 2 | 3 | 4 | |
|---|---|---|---|---|---|
| No i | ◯ | ◯ | ◯ | ◯ | All items are relevant |

18. Comprehensiveness *

Does this tool miss any key aspects of your experience of fatigue in DCM?

*Mark only one oval.*

| | 1 | 2 | 3 | 4 | |
|---|---|---|---|---|---|
| Key | ◯ | ◯ | ◯ | ◯ | No key aspects are missing |

19. Clarity *

Was this tool clear to understand?

*Mark only one oval.*

| | 1 | 2 | 3 | 4 | |
|---|---|---|---|---|---|
| Not | ◯ | ◯ | ◯ | ◯ | Very clear |

20. How long you do you think it would take to fill this tool? *

Answer in minutes

_______________________________

21. Comments

## Tool #5

### Multidimensional fatigue inventory (MFI)

Please take a look at the tool below and answer the four questions:

# MFI® MULTIDIMENSIONAL FATIGUE INVENTORY

® E. Smets, B.Garssen, B. Bonke.

**Instructions:**

By means of the following statements we would like to get an idea of how you have been feeling **lately**. There is, for example, the statement:

"I FEEL RELAXED"

If you think that this is **entirely true**, that indeed you have been feeling relaxed lately, please, place an **X** in the extreme left box; like this:

**yes, that is true ☒1 ❑2 ❑3 ❑4 ❑5 no, that is not true**

The more you **disagree** with the statement, the more you can place an **X** in the direction of "no, that is not true". Please do not miss out a statement and place only one **X** in a box for each statement.

| | | | | | | | | |
|---|---|---|---|---|---|---|---|---|
| 1 | I feel fit. | yes, that is true | ❑1 | ❑2 | ❑3 | ❑4 | ❑5 | no, that is not true |
| 2 | Physically, I feel only able to do a little. | yes, that is true | ❑1 | ❑2 | ❑3 | ❑4 | ❑5 | no, that is not true |
| 3 | I feel very active. | yes, that is true | ❑1 | ❑2 | ❑3 | ❑4 | ❑5 | no, that is not true |
| 4 | I feel like doing all sorts of nice things. | yes, that is true | ❑1 | ❑2 | ❑3 | ❑4 | ❑5 | no, that is not true |
| 5 | I feel tired. | yes, that is true | ❑1 | ❑2 | ❑3 | ❑4 | ❑5 | no, that is not true |
| 6 | I think I do a lot in a day. | yes, that is true | ❑1 | ❑2 | ❑3 | ❑4 | ❑5 | no, that is not true |
| 7 | When I am doing something, I can keep my thoughts on it. | yes, that is true | ❑1 | ❑2 | ❑3 | ❑4 | ❑5 | no, that is not true |
| 8 | Physically I can take on a lot. | yes, that is true | ❑1 | ❑2 | ❑3 | ❑4 | ❑5 | no, that is not true |
| 9 | I dread having to do things. | yes, that is true | ❑1 | ❑2 | ❑3 | ❑4 | ❑5 | no, that is not true |
| 10 | I think I do very little in a day. | yes, that is true | ❑1 | ❑2 | ❑3 | ❑4 | ❑5 | no, that is not true |
| 11 | I can concentrate well. | yes, that is true | ❑1 | ❑2 | ❑3 | ❑4 | ❑5 | no, that is not true |
| 12 | I am rested. | yes, that is true | ❑1 | ❑2 | ❑3 | ❑4 | ❑5 | no, that is not true |
| 13 | It takes a lot of effort to concentrate on things. | yes, that is true | ❑1 | ❑2 | ❑3 | ❑4 | ❑5 | no, that is not true |
| 14 | Physically I feel I am in a bad condition. | yes, that is true | ❑1 | ❑2 | ❑3 | ❑4 | ❑5 | no, that is not true |
| 15 | I have a lot of plans. | yes, that is true | ❑1 | ❑2 | ❑3 | ❑4 | ❑5 | no, that is not true |
| 16 | I tire easily. | yes, that is true | ❑1 | ❑2 | ❑3 | ❑4 | ❑5 | no, that is not true |
| 17 | I get little done. | yes, that is true | ❑1 | ❑2 | ❑3 | ❑4 | ❑5 | no, that is not true |
| 18 | I don't feel like doing anything. | yes, that is true | ❑1 | ❑2 | ❑3 | ❑4 | ❑5 | no, that is not true |
| 19 | My thoughts easily wander. | yes, that is true | ❑1 | ❑2 | ❑3 | ❑4 | ❑5 | no, that is not true |
| 20 | Physically I feel I am in an excellent condition. | yes, that is true | ❑1 | ❑2 | ❑3 | ❑4 | ❑5 | no, that is not true |

22. Relevance to DCM *

Are all items in this tool relevant to your experience of fatigue in DCM?

*Mark only one oval.*

| | 1 | 2 | 3 | 4 | |
|---|---|---|---|---|---|
| No i | ◯ | ◯ | ◯ | ◯ | All items are relevant |

23. Comprehensiveness *

Does this tool miss any key aspects of your experience of fatigue in DCM?

*Mark only one oval.*

| | 1 | 2 | 3 | 4 | |
|---|---|---|---|---|---|
| Key | ◯ | ◯ | ◯ | ◯ | No key aspects are missing |

24. Clarity *

Was this tool clear to understand?

*Mark only one oval.*

| | 1 | 2 | 3 | 4 | |
|---|---|---|---|---|---|
| Not | ◯ | ◯ | ◯ | ◯ | Very clear |

25. How long you do you think it would take to fill this tool? *

Answer in minutes

_______________________________

26. Comments

________________________________
________________________________
________________________________
________________________________
________________________________

Tool #6

Numeric rating scale: fatigue

Please take a look at the tool below and answer the four questions:

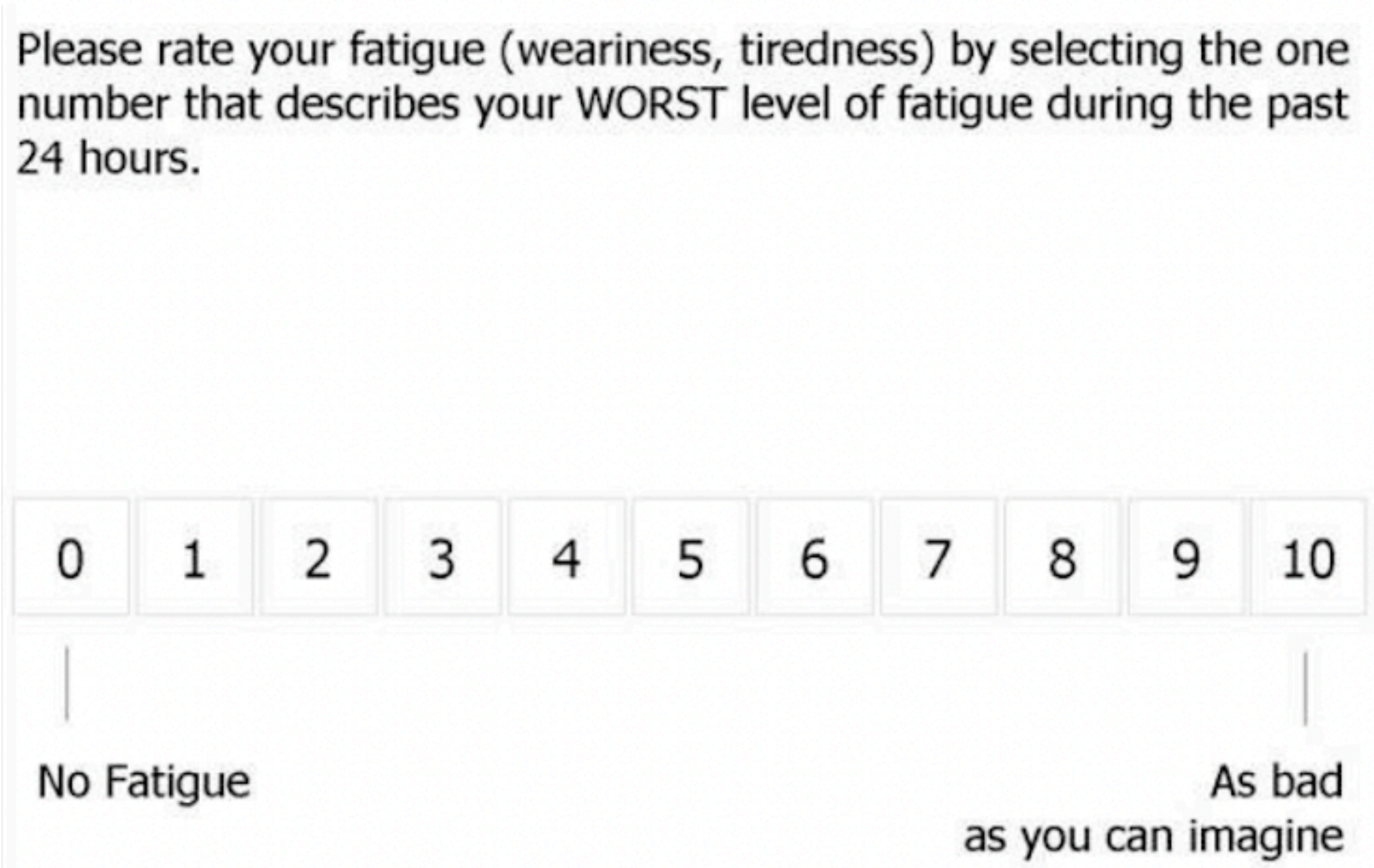


27. Relevance to DCM *

Are all items in this tool relevant to your experience of fatigue in DCM?

*Mark only one oval.*

| | 1 | 2 | 3 | 4 | |
|---|---|---|---|---|---|
| No i | ◯ | ◯ | ◯ | ◯ | All items are relevant |

28. Comprehensiveness *

Does this tool miss any key aspects of your experience of fatigue in DCM?

*Mark only one oval.*

| | 1 | 2 | 3 | 4 | |
|---|---|---|---|---|---|
| Key | ◯ | ◯ | ◯ | ◯ | No key aspects are missing |

29. Clarity *

Was this tool clear to understand?

*Mark only one oval.*

| | 1 | 2 | 3 | 4 | |
|---|---|---|---|---|---|
| Not | ◯ | ◯ | ◯ | ◯ | Very clear |

30. How long you do you think it would take to fill this tool? *

Answer in minutes

_______________________

31. Comments

_______________________

_______________________

_______________________

_______________________

_______________________

## Tool #7

Visual analogue scale for fatigue (VAS-F)

Please take a look at the tool below and answer the four questions:

Click to view:

https://drive.google.com/file/d/1n1LKogYThSGYNxK0ZsuI6v8IgDqSCCo4/view?usp=sharing

32. Relevance to DCM *

Are all items in this tool relevant to your experience of fatigue in DCM?

*Mark only one oval.*

| | 1 | 2 | 3 | 4 | |
|---|---|---|---|---|---|
| No i | ◯ | ◯ | ◯ | ◯ | All items are relevant |

33. Comprehensiveness *

Does this tool miss any key aspects of your experience of fatigue in DCM?

*Mark only one oval.*

| | 1 | 2 | 3 | 4 | |
|---|---|---|---|---|---|
| Key | ◯ | ◯ | ◯ | ◯ | No key aspects are missing |

34. Clarity *

Was this tool clear to understand?

*Mark only one oval.*

| | 1 | 2 | 3 | 4 | |
|---|---|---|---|---|---|
| Not | ◯ | ◯ | ◯ | ◯ | Very clear |

35. How long you do you think it would take to fill this tool? *

Answer in minutes

_______________________________

36. Comments

Tool #8

36-item short form health survey: fatigue subscale (SF-36-Fatigue)
Please take a look at the tool below and answer the four questions:

Click to view:
https://drive.google.com/file/d/1G7D9vye6pM_zHAUBPus1NdMeN0jDAPy3/view?usp=sharing

37. Relevance to DCM *

Are all items in this tool relevant to your experience of fatigue in DCM?

*Mark only one oval.*

| | 1 | 2 | 3 | 4 | |
|---|---|---|---|---|---|
| No i | ◯ | ◯ | ◯ | ◯ | All items are relevant |

38. Comprehensiveness *

Does this tool miss any key aspects of your experience of fatigue in DCM?

*Mark only one oval.*

| | 1 | 2 | 3 | 4 | |
|---|---|---|---|---|---|
| Key | ◯ | ◯ | ◯ | ◯ | No key aspects are missing |

39. Clarity *

Was this tool clear to understand?

*Mark only one oval.*

| | 1 | 2 | 3 | 4 | |
|---|---|---|---|---|---|
| Not | ◯ | ◯ | ◯ | ◯ | Very clear |

40. How long you do you think it would take to fill this tool? *

Answer in minutes

________________________________

41. Comments

________________________________________________________________

________________________________________________________________

________________________________________________________________

________________________________________________________________

________________________________________________________________

## Tool #9

Fatigue symptom inventory (FSI)

Please take a look at the tool below and answer the four questions:

Click to view:
[https://drive.google.com/file/d/11SubXQ1i3AIgeRqVeZy6cNMiGpAP5dyU/view?usp=sharing](https://drive.google.com/file/d/11SubXQ1i3AIgeRqVeZy6cNMiGpAP5dyU/view?usp=sharing)

42. Relevance to DCM *

Are all items in this tool relevant to your experience of fatigue in DCM?

*Mark only one oval.*

| | 1 | 2 | 3 | 4 | |
|---|---|---|---|---|---|
| No i | ◯ | ◯ | ◯ | ◯ | All items are relevant |

43. Comprehensiveness *

Does this tool miss any key aspects of your experience of fatigue in DCM?

*Mark only one oval.*

| | 1 | 2 | 3 | 4 | |
|---|---|---|---|---|---|
| Key | ◯ | ◯ | ◯ | ◯ | No key aspects are missing |

44. Clarity *

Was this tool clear to understand?

*Mark only one oval.*

| | 1 | 2 | 3 | 4 | |
|---|---|---|---|---|---|
| Not | ◯ | ◯ | ◯ | ◯ | Very clear |

45. How long you do you think it would take to fill this tool? *

Answer in minutes

_______________________________

46. Comments

_______________________________
_______________________________
_______________________________
_______________________________
_______________________________

## Tool #10

### Fatigue assessment instrument (FAI)

Please take a look at the tool below and answer the four questions:

**Fatigue Assessment Inventory**

Instructions:

Below are a series of statements regarding your Fatigue. By Fatigue we mean a sense of tiredness, lack of energy or total body give-out. Please read each statement and choose a number from 1 to 7, where #1 indicates you completely disagree with the statement and #7 indicates you completely agree. Please answer these questions as they apply to the past TWO WEEKS.

Circle the appropriate number on the answer sheet!

*Questions:*

| | Completely Disagree | | | | | | Completely agree |
|---|---|---|---|---|---|---|---|
| 1. I feel drowsy when I am fatigued. | 1 | 2 | 3 | 4 | 5 | 6 | 7 |
| 2. When I am fatigued, I lose my patience. | 1 | 2 | 3 | 4 | 5 | 6 | 7 |
| 3. My motivation is lower when I am fatigued. | 1 | 2 | 3 | 4 | 5 | 6 | 7 |
| 4. When I am fatigued, I have difficulty concentrating. | 1 | 2 | 3 | 4 | 5 | 6 | 7 |
| 5. Exercise brings on my fatigue. | 1 | 2 | 3 | 4 | 5 | 6 | 7 |
| 6. Heat brings on my fatigue. | 1 | 2 | 3 | 4 | 5 | 6 | 7 |
| 7. Long periods of inactivity bring on my fatigue | 1 | 2 | 3 | 4 | 5 | 6 | 7 |
| 8. Stress brings on my fatigue. | 1 | 2 | 3 | 4 | 5 | 6 | 7 |
| 9. Depression brings on my fatigue. | 1 | 2 | 3 | 4 | 5 | 6 | 7 |
| 10. Work brings on fatigue. | 1 | 2 | 3 | 4 | 5 | 6 | 7 |
| 11. My fatigue is worse in the afternoon. | 1 | 2 | 3 | 4 | 5 | 6 | 7 |
| 12. My fatigue is worse in the morning. | 1 | 2 | 3 | 4 | 5 | 6 | 7 |
| 13. Performance of routine daily activities increases my fatigue. | 1 | 2 | 3 | 4 | 5 | 6 | 7 |
| 14. Resting lessens my fatigue. | 1 | 2 | 3 | 4 | 5 | 6 | 7 |
| 15. Sleeping lessens my fatigue. | 1 | 2 | 3 | 4 | 5 | 6 | 7 |
| 16. Cool temperatures lessen my fatigue. | 1 | 2 | 3 | 4 | 5 | 6 | 7 |
| 17. Positive experiences lessen my fatigue. | 1 | 2 | 3 | 4 | 5 | 6 | 7 |
| 18. I am easily fatigued. | 1 | 2 | 3 | 4 | 5 | 6 | 7 |
| 19. Fatigue interferes with my physical functioning. | 1 | 2 | 3 | 4 | 5 | 6 | 7 |
| 20. Fatigue causes frequent problems for me. | 1 | 2 | 3 | 4 | 5 | 6 | 7 |
| 21. My fatigue prevents sustained physical functioning. | 1 | 2 | 3 | 4 | 5 | 6 | 7 |
| 22. Fatigue interferes with carrying out certain duties and responsibilities. | 1 | 2 | 3 | 4 | 5 | 6 | 7 |
| 23. Fatigue predated other symptoms of my condition. | 1 | 2 | 3 | 4 | 5 | 6 | 7 |
| 24. Fatigue is my most disabling symptom | 1 | 2 | 3 | 4 | 5 | 6 | 7 |
| 25. Fatigue is among my 3 most disabling symptoms. | 1 | 2 | 3 | 4 | 5 | 6 | 7 |
| 26. Fatigue interferes with my work, family or social life. | 1 | 2 | 3 | 4 | 5 | 6 | 7 |
| 27. Fatigue makes other symptoms worse. | 1 | 2 | 3 | 4 | 5 | 6 | 7 |
| 28. Fatigue that I now experience is different in quality or severity than the fatigue I experienced before I developed this condition | 1 | 2 | 3 | 4 | 5 | 6 | 7 |
| 29. I experienced prolonged fatigue after exercise. | 1 | 2 | 3 | 4 | 5 | 6 | 7 |

47. Relevance to DCM *

Are all items in this tool relevant to your experience of fatigue in DCM?

*Mark only one oval.*

| | 1 | 2 | 3 | 4 | |
|---|---|---|---|---|---|
| No i | ◯ | ◯ | ◯ | ◯ | All items are relevant |

48. Comprehensiveness *

Does this tool miss any key aspects of your experience of fatigue in DCM?

*Mark only one oval.*

| | 1 | 2 | 3 | 4 | |
|---|---|---|---|---|---|
| Key | ◯ | ◯ | ◯ | ◯ | No key aspects are missing |

49. Clarity *

Was this tool clear to understand?

*Mark only one oval.*

| | 1 | 2 | 3 | 4 | |
|---|---|---|---|---|---|
| Not | ◯ | ◯ | ◯ | ◯ | Very clear |

50. How long you do you think it would take to fill this tool? *

Answer in minutes

_______________________________

51. Comments

Tool #11

Brief fatigue inventory (BFI)

Please take a look at the tool below and answer the four questions:

# Brief Fatigue Inventory

STUDY ID# ____________ HOSPITAL # ____________

**Date:** ______/______/______ **Time:** ____________

**Name** ____________________ ____________________ ____________
Last First Middle Initial

**Throughout our lives, most of us have times when we feel very tired or fatigued. Have you felt unusually tired or fatigued in the last week? Yes ☐ No ☐**

**1. Please rate your fatigue (weariness, tiredness) by circling the one number that best describes your fatigue right NOW.**

0 1 2 3 4 5 6 7 8 9 10
No Fatigue — As bad as you can imagine

**2. Please rate your fatigue (weariness, tiredness) by circling the one number that best describes your USUAL level of fatigue during past 24 hours.**

0 1 2 3 4 5 6 7 8 9 10
No Fatigue — As bad as you can imagine

**3. Please rate your fatigue (weariness, tiredness) by circling the one number that best describes your WORST level of fatigue during past 24 hours.**

0 1 2 3 4 5 6 7 8 9 10
No Fatigue — As bad as you can imagine

**4. Circle the one number that describes how, during the past 24 hours, fatigue has interfered with your:**

**A. General activity**
0 1 2 3 4 5 6 7 8 9 10
Does not interfere — Completely Interferes

**B. Mood**
0 1 2 3 4 5 6 7 8 9 10
Does not interfere — Completely Interferes

**C. Walking ability**
0 1 2 3 4 5 6 7 8 9 10
Does not interfere — Completely Interferes

**D. Normal work (includes both work outside the home and daily chores)**
0 1 2 3 4 5 6 7 8 9 10
Does not interfere — Completely Interferes

**E. Relations with other people**
0 1 2 3 4 5 6 7 8 9 10
Does not interfere — Completely Interferes

**F. Enjoyment of life**
0 1 2 3 4 5 6 7 8 9 10
Does not interfere — Completely Interferes

52. Relevance to DCM *

Are all items in this tool relevant to your experience of fatigue in DCM?

*Mark only one oval.*

| | 1 | 2 | 3 | 4 | |
|---|---|---|---|---|---|
| No i | ◯ | ◯ | ◯ | ◯ | All items are relevant |

53. Comprehensiveness *

Does this tool miss any key aspects of your experience of fatigue in DCM?

*Mark only one oval.*

| | 1 | 2 | 3 | 4 | |
|---|---|---|---|---|---|
| Key | ◯ | ◯ | ◯ | ◯ | No key aspects are missing |

54. Clarity *

Was this tool clear to understand?

*Mark only one oval.*

| | 1 | 2 | 3 | 4 | |
|---|---|---|---|---|---|
| Not | ◯ | ◯ | ◯ | ◯ | Very clear |

55. How long you do you think it would take to fill this tool? *

Answer in minutes

_______________________

56. Comments

Tool #12

Piper fatigue scale (PFS)

Please take a look at the tool below and answer the four questions:

Click to view:
https://drive.google.com/file/d/1uQOuQ7DjqUyKNS8QZSwUWt8xetHzO_C0/view?usp=sharing

57. Relevance to DCM *

Are all items in this tool relevant to your experience of fatigue in DCM?

*Mark only one oval.*

| | 1 | 2 | 3 | 4 | |
|---|---|---|---|---|---|
| No i | ◯ | ◯ | ◯ | ◯ | All items are relevant |

58. Comprehensiveness *

Does this tool miss any key aspects of your experience of fatigue in DCM?

*Mark only one oval.*

| | 1 | 2 | 3 | 4 | |
|---|---|---|---|---|---|
| Key | ◯ | ◯ | ◯ | ◯ | No key aspects are missing |

59. Clarity *

Was this tool clear to understand?

*Mark only one oval.*

| | 1 | 2 | 3 | 4 | |
|---|---|---|---|---|---|
| Not | ◯ | ◯ | ◯ | ◯ | Very clear |

60. How long you do you think it would take to fill this tool? *

Answer in minutes

____________________________________

61. Comments

______________________________________________________________________

______________________________________________________________________

______________________________________________________________________

______________________________________________________________________

______________________________________________________________________

## Tool #13

Chalder fatigue scale (CFS)

Please take a look at the tool below and answer the four questions:

Click to view:
https://drive.google.com/file/d/1zgWMmUstkxGQrWii48hyCNJiwDnptMuz/view?usp=sharing

62. Relevance to DCM *

Are all items in this tool relevant to your experience of fatigue in DCM?

*Mark only one oval.*

| | 1 | 2 | 3 | 4 | |
|---|---|---|---|---|---|
| No i | ◯ | ◯ | ◯ | ◯ | All items are relevant |

63. Comprehensiveness *

Does this tool miss any key aspects of your experience of fatigue in DCM?

*Mark only one oval.*

| | 1 | 2 | 3 | 4 | |
|---|---|---|---|---|---|
| Key | ◯ | ◯ | ◯ | ◯ | No key aspects are missing |

64. Clarity *

Was this tool clear to understand?

*Mark only one oval.*

| | 1 | 2 | 3 | 4 | |
|---|---|---|---|---|---|
| Not | ◯ | ◯ | ◯ | ◯ | Very clear |

65. How long you do you think it would take to fill this tool? *

Answer in minutes

_______________________________

66. Comments

_______________________________
_______________________________
_______________________________
_______________________________
_______________________________

## Tool #14

Chronic fatigue syndrome-activities and participation questionnaire (CFS-APQ)
Please take a look at the tool below and answer the four questions:

Click to view:
https://drive.google.com/file/d/1M7dXT1wTy2ByQrQ1eyZrZyAaK_2C0PSP/view?usp=sharing

67. Relevance to DCM *

Are all items in this tool relevant to your experience of fatigue in DCM?

*Mark only one oval.*

| | 1 | 2 | 3 | 4 | |
|---|---|---|---|---|---|
| No i | ◯ | ◯ | ◯ | ◯ | All items are relevant |

68. Comprehensiveness *

Does this tool miss any key aspects of your experience of fatigue in DCM?

*Mark only one oval.*

| | 1 | 2 | 3 | 4 | |
|---|---|---|---|---|---|
| Key | ◯ | ◯ | ◯ | ◯ | No key aspects are missing |

69. Clarity *

Was this tool clear to understand?

*Mark only one oval.*

| | 1 | 2 | 3 | 4 | |
|---|---|---|---|---|---|
| Not | ◯ | ◯ | ◯ | ◯ | Very clear |

70. How long you do you think it would take to fill this tool? *

Answer in minutes

_______________________________

71. Comments

## Tool #15

Fatigue assessment scale (FAS)

Please take a look at the tool below and answer the four questions:

Fatigue Assessment Scale (FAS)

The following 10 statements refer to how you usually feel. For each statement you can choose one out of five answer categories, varying from *never* to *always*. 1 = *never*; 2 = *sometimes;* 3 = *regularly*; 4 = *often*; 5 = *always*.

| | Never | Sometimes | Regularly | Often | Always |
|---|---|---|---|---|---|
| 1. I am bothered by fatigue (WHOQOL) | 1 | 2 | 3 | 4 | 5 |
| 2. I get tired very quickly (CIS) | 1 | 2 | 3 | 4 | 5 |
| 3. I don't do much during the day (CIS) | 1 | 2 | 3 | 4 | 5 |
| 4. I have enough energy for everyday life (WHOQOL) | 1 | 2 | 3 | 4 | 5 |
| 5. Physically, I feel exhausted (CIS) | 1 | 2 | 3 | 4 | 5 |
| 6. I have problems starting things (FS) | 1 | 2 | 3 | 4 | 5 |
| 7. I have problems thinking clearly (FS) | 1 | 2 | 3 | 4 | 5 |
| 8. I feel no desire to do anything (CIS) | 1 | 2 | 3 | 4 | 5 |
| 9. Mentally, I feel exhausted | 1 | 2 | 3 | 4 | 5 |
| 10. When I am doing something, I can concentrate quite well (CIS) | 1 | 2 | 3 | 4 | 5 |

72. Relevance to DCM *

Are all items in this tool relevant to your experience of fatigue in DCM?

*Mark only one oval.*

| | 1 | 2 | 3 | 4 | |
|---|---|---|---|---|---|
| No i | ◯ | ◯ | ◯ | ◯ | All items are relevant |

73. Comprehensiveness *

Does this tool miss any key aspects of your experience of fatigue in DCM?

*Mark only one oval.*

| | 1 | 2 | 3 | 4 | |
|---|---|---|---|---|---|
| Key | ◯ | ◯ | ◯ | ◯ | No key aspects are missing |

74. Clarity *

Was this tool clear to understand?

*Mark only one oval.*

| | 1 | 2 | 3 | 4 | |
|---|---|---|---|---|---|
| Not | ◯ | ◯ | ◯ | ◯ | Very clear |

75. How long you do you think it would take to fill this tool? *

Answer in minutes

_______________________

76. Comments

_______________________
_______________________
_______________________
_______________________
_______________________

## Tool #16

### Checklist individual strength (CIS)

Please take a look at the tool below and answer the four questions:

**Instruction:**

On the next page you find 20 statements. With these statements we wish to get an impression of how you have felt during the past two weeks. For example:

**I feel relaxed**

If you feel that this statement is not true at all, place a cross in the right box; like this:

| | | | | | | | | | |
|---|---|---|---|---|---|---|---|---|---|
| I feel relaxed | **yes**, that is true | X | | | | | | | **no**, that is not true |

If you feel that this statement is not true at all, place a cross in the right box; like this:

| | | | | | | | | | |
|---|---|---|---|---|---|---|---|---|---|
| I feel relaxed | **yes**, that is true | | | | | | | X | **no**, that is not true |

If you feel that this statement in not "yes, that is true", but also not "no, that is not true", place a cross in the box that is most in accordance with how you have felt.

For example, if you feel relaxed, but not very relaxed, place a cross in one of the boxes close to "yes, that is true": like this:

| | | | | | | | | | |
|---|---|---|---|---|---|---|---|---|---|
| I feel relaxed | **yes**, that is true | | | X | | | | | **no**, that is not true |

Do not skip any statement and place only one cross for each statement.

| | | | | | | | | | |
|---|---|---|---|---|---|---|---|---|---|
| 1. I feel tired | yes, that is true | | | | | | | | no, that is not true |
| 2. I feel very active | yes, that is true | | | | | | | | no, that is not true |
| 3. Thinking requires effort | yes, that is true | | | | | | | | no, that is not true |
| 4. Physically I feel exhausted | yes, that is true | | | | | | | | no, that is not true |
| 5. I feel like doing all kinds of nice things | yes, that is true | | | | | | | | no, that is not true |
| 6. I feel fit | yes, that is true | | | | | | | | no, that is not true |
| 7. I do quite a lot within a day | yes, that is true | | | | | | | | no, that is not true |
| 8. When I am doing something, I can concentrate quite well | yes, that is true | | | | | | | | no, that is not true |
| 9. I feel weak | yes, that is true | | | | | | | | no, that is not true |
| 10. I don't do much during the day | yes, that is true | | | | | | | | no, that is not true |
| 11. I can concentrate well | yes, that is true | | | | | | | | no, that is not true |
| 12. I feel rested | yes, that is true | | | | | | | | no, that is not true |
| 13. I have trouble concentrating | yes, that is true | | | | | | | | no, that is not true |
| 14. Physically I feel I am in a bad condition | yes, that is true | | | | | | | | no, that is not true |
| 15. I am full of plans | yes, that is true | | | | | | | | no, that is not true |
| 16. I get tired very quickly | yes, that is true | | | | | | | | no, that is not true |
| 17. I have a low output | yes, that is true | | | | | | | | no, that is not true |
| 18. I feel no desire to do anything | yes, that is true | | | | | | | | no, that is not true |
| 19. My thoughts easily wander | yes, that is true | | | | | | | | no, that is not true |
| 20. Physically I feel in a good shape | yes, that is true | | | | | | | | no, that is not true |

**SCORING CIS20R**

For the items: **2, 5, 6, 7, 8, 11, 12, 15, 20** is the scoring as follows:

| | | | | | | | | |
|---|---|---|---|---|---|---|---|---|
| **yes**, that is true | 1 | 2 | 3 | 4 | 5 | 6 | 7 | **no**, that is not true |

For the items: **1, 3, 4, 9, 10, 13, 14, 16, 17, 18, 19** is the scoring as follows:

| | | | | | | | | |
|---|---|---|---|---|---|---|---|---|
| **yes**, that is true | 7 | 6 | 5 | 4 | 3 | 2 | 1 | **no**, that is not true |

Subsequently **the four subscales** are calculated by summing the respective items

| | |
|---|---|
| **subscale 1: Subjective feeling of fatigue** | items 1, 4, 6, 9, 12, 14, 16, 20 |
| **subscale 2: Concentration** | items 3, 8, 11, 13, 19 |
| **subscale 3: Motivation** | items 2, 5, 15, 18 |
| **subscale 4: Physical activity** | items 7, 10, 17 |

77. Relevance to DCM *

Are all items in this tool relevant to your experience of fatigue in DCM?

*Mark only one oval.*

| | 1 | 2 | 3 | 4 | |
|---|---|---|---|---|---|
| No i | ◯ | ◯ | ◯ | ◯ | All items are relevant |

78. Comprehensiveness *

Does this tool miss any key aspects of your experience of fatigue in DCM?

*Mark only one oval.*

| | 1 | 2 | 3 | 4 | |
|---|---|---|---|---|---|
| Key | ◯ | ◯ | ◯ | ◯ | No key aspects are missing |

79. Clarity *

Was this tool clear to understand?

*Mark only one oval.*

| | 1 | 2 | 3 | 4 | |
|---|---|---|---|---|---|
| Not | ◯ | ◯ | ◯ | ◯ | Very clear |

80. How long you do you think it would take to fill this tool? *

Answer in minutes

_______________________________

81. Comments

## Tool #17

Bristol rheumatoid arthritis fatigue: multidimensional questionnaire (BRAF-MDQ)
Please take a look at the tool below and answer the four questions:

Click to view:
https://drive.google.com/file/d/1ud1ptaBabodnToICW4-3dhb2MwuaxQq_/view?usp=sharing

82. Relevance to DCM *

Are all items in this tool relevant to your experience of fatigue in DCM?

*Mark only one oval.*

| | 1 | 2 | 3 | 4 | |
|---|---|---|---|---|---|
| No i | ◯ | ◯ | ◯ | ◯ | All items are relevant |

83. Comprehensiveness *

Does this tool miss any key aspects of your experience of fatigue in DCM?

*Mark only one oval.*

| | 1 | 2 | 3 | 4 | |
|---|---|---|---|---|---|
| Key | ◯ | ◯ | ◯ | ◯ | No key aspects are missing |

84. Clarity *

Was this tool clear to understand?

*Mark only one oval.*

| | 1 | 2 | 3 | 4 | |
|---|---|---|---|---|---|
| Not | ◯ | ◯ | ◯ | ◯ | Very clear |

85. How long you do you think it would take to fill this tool? *

Answer in minutes

_______________________

86. Comments

_______________________
_______________________
_______________________
_______________________
_______________________

Tool #18

Fibrofatigue scale (FFS)

Please take a look at the tool below and answer the four questions:

Click to view:
https://drive.google.com/file/d/1u-J5dpaseAHGoRnWW0dkK1mjvUd6xkvb/view?usp=sharing

87. Relevance to DCM *

Are all items in this tool relevant to your experience of fatigue in DCM?

*Mark only one oval.*

| | 1 | 2 | 3 | 4 | |
|---|---|---|---|---|---|
| No i | ◯ | ◯ | ◯ | ◯ | All items are relevant |

88. Comprehensiveness *

Does this tool miss any key aspects of your experience of fatigue in DCM?

*Mark only one oval.*

| | 1 | 2 | 3 | 4 | |
|---|---|---|---|---|---|
| Key | ◯ | ◯ | ◯ | ◯ | No key aspects are missing |

89. Clarity *

Was this tool clear to understand?

*Mark only one oval.*

| | 1 | 2 | 3 | 4 | |
|---|---|---|---|---|---|
| Not | ◯ | ◯ | ◯ | ◯ | Very clear |

90. How long you do you think it would take to fill this tool? *

Answer in minutes

_______________

91. Comments

_______________
_______________
_______________
_______________
_______________

Tool #19

Multidimensional daily diary of fatigue-fibromyalgia-17 items (MDF-fibro-17)

Please take a look at the tool below and answer the four questions:

'On a scale of 0 to 10...' (0 being the lowest and 10 being the highest)

1. How severe was your fatigue today?
2. How worn out did you feel today?
3. How easily did you get tired today?
4. How exhausted did you feel today?

5. How weak did your muscles feel today?
6. How heavy did your body feel today?
7. How tired did your body feel today?

8. How difficult was it to concentrate because you were tired today?
9. How much did tiredness make it difficult to think clearly today?
10. How much did tiredness make it difficult to remember today?
11. How difficult was it to focus today?

12. How much of an effort was it to do things today?
13. How much did you have to force yourself to do things?
14. How difficult was it to get motivated to do things today?

15. How much did tiredness make it difficult to do things today?
16. Did it take you longer to do things today because you were tired?
17. Did you do things more slowly because you

were tired today?

92. Relevance to DCM *

Are all items in this tool relevant to your experience of fatigue in DCM?

*Mark only one oval.*

| | 1 | 2 | 3 | 4 | |
|---|---|---|---|---|---|
| No i | ◯ | ◯ | ◯ | ◯ | All items are relevant |

93. Comprehensiveness *

Does this tool miss any key aspects of your experience of fatigue in DCM?

*Mark only one oval.*

| | 1 | 2 | 3 | 4 | |
|---|---|---|---|---|---|
| Key | ◯ | ◯ | ◯ | ◯ | No key aspects are missing |

94. Clarity *

Was this tool clear to understand?

*Mark only one oval.*

| | 1 | 2 | 3 | 4 | |
|---|---|---|---|---|---|
| Not | ◯ | ◯ | ◯ | ◯ | Very clear |

95. How long you do you think it would take to fill this tool? *

Answer in minutes

_______________________

96. Comments

Confirm your submission

This content is neither created nor endorsed by Google.

Google Forms
